# Current and future economic burden of diabetes among working-age adults in Asia: conservative estimates for Singapore from 2010-2050

**DOI:** 10.1186/s12889-016-2827-1

**Published:** 2016-02-16

**Authors:** May Ee Png, Joanne Yoong, Thao Phuong Phan, Hwee Lin Wee

**Affiliations:** National University of Singapore, Saw Swee Hock School of Public Health, Singapore, Singapore; University of Southern California, Center for Economic and Social Research, Los Angeles, USA; Department of Pharmacy, National University of Singapore, Singapore, Singapore

**Keywords:** Diabetes, Working-age, Projection, Cost, Singapore

## Abstract

**Background:**

Diabetes not only imposes a huge health burden but also a large economic burden worldwide. In the working-age population, cost of lost productivity can far exceed diabetes-related medical cost. In this study, we aimed to estimate the current and future indirect and excess direct costs of diagnosed type 2 diabetes among the working-age population in Singapore.

**Methods:**

A previously-published epidemiological model of diabetes was adapted to forecast prevalence among working-age patients with diagnosed type 2 diabetes in the absence of interventions. The current methodology of the American Diabetes Association was adopted to estimate the costs of diabetes for this population. Diabetes-related excess direct medical costs were obtained from a local cost study while indirect costs were calculated using the human capital approach applied to local labor force statistics. These cost were estimated conservatively from a societal perspective on a per patient basis and projected to the overall Singapore population from 2010 to 2050.

**Results:**

In 2010, total economic costs per working-age patient were estimated to be US$5,646 (US$4,432-US$10,612), of which 42 % were excess direct medical costs and 58 % indirect productivity-related losses. Total cost is projected to rise to US$7,791 (US$5,741-US$12,756) in 2050, with the share of indirect costs rising to 65 %. Simultaneous increases in prevalence imply that the total economic costs of diabetes for the entire working-age population will increase by 2.4 fold from US$787 million in 2010 to US$1,867 million in 2050.

**Conclusions:**

By current projections, diabetes in Singapore represents a growing economic burden. Among the working-age population, the impact of productivity loss will become increasingly significant. Prevention efforts to reduce overall prevalence should also engage stakeholders outside the health sector who ultimately bear the indirect burden of disease.

## Background

Type 2 diabetes mellitus is a chronic metabolic disease which results not only in significant direct medical costs but indirect productivity losses due to disability and early mortality. For this reason, the future growth of type 2 diabetes is an increasing concern to researchers worldwide: in the U.S. for instance, type 2 diabetes is projected to almost double from a disease population of 23.7 million in 2009 to 44.1 million in 2034, resulting in a more-than-proportional tripling of diabetes-related spending from US$113 billion to US$336 billion [1]. However, the composition of social costs varies significantly by population subgroups: while many analyses focus on the direct cost burden for older and retired individuals, in the working-age population, lost productivity can far exceed disease-related spending [2].

To date, there have been few comprehensive analyses of current and future diabetes-related social costs in Asia, notably for Singapore. Globally, studies of national economic burden such as the American Diabetes Association (ADA) studies have typically been cross-sectional, focusing on the retrospective assessment of costs in a given year. At the same time, efforts to incorporate projection of costs from a societal perspective for the diabetes population have been observed in studies conducted in Australia, Canada, China, Colombia, Iran, IMS Health as well as the latest study from U.K. [3–7]. In Singapore, the best available current prediction from the International Diabetes Federation (IDF) suggests health expenditures of US$0.83–1.48 billion in 2030 for Singapore. However, this figure is an aggregate estimate based on a cross-country model with no insight into potential indirect costs [8]. The lack of comprehensive social cost studies for Asia is critical for regional decision makers, since comparison of national health expenditure is complicated by differences in disease epidemiology as well as healthcare financing systems [9].

It is also critical to understand the changing dynamics of disease and costs: for instance, Asian populations are currently at higher risk of developing type 2 diabetes compared to others [10], but are also seeing trends of earlier disease onset that may have strong implications for overall economic growth and employment.

In this study, we focus our attention on estimating the current and future total economic burden of diagnosed diabetes for the working-age population (i.e., 20–69 years old) in Singapore, which has the highest proportion of younger patients in the region and is also aging rapidly [11]. We provide estimates of the composition of indirect and excess direct costs among this population, to assess potential justification for interventions within as well as beyond the health sector. We then project the estimated costs into the future by adopting the latest methodology described by the ADA, as well as using a published local micro-simulation disease model, the Demographic Epidemiological Model of Singapore (DEMOS) [12, 13].

## Methods

### Prevalence of diabetes in the workforce

In this model, the total number of diabetes patients in the workforce was estimated by combining age- and gender-specific population totals, workforce participation rates α, and diabetes prevalence rates β, to arrive at age-gender–specific totals for the employed population as well as the total number of diabetes patients in the general population. The age and gender-specific prevalence of diagnosed diabetes in the population was obtained from previously-reported prevalence estimates based on the Singapore Prospective Study (SP2) and the Singapore National Health Survey [13] and further assuming a 50 % undiagnosed rate as reported in the 2010 National Health Survey [14]. In order to take account of the importance of gender differences in both labor force participation patterns and the epidemiology of diabetes, gender-disaggregated data was used whenever available and relevant in our analysis. Overall workforce population in Singapore by gender and age (in ten-year bands) from 20 to 69 years old was obtained from the 2010 census and data from the Ministry of Manpower [15–18].

Based on the estimated difference in absolute probability of employment for diabetes patient versus individuals without diabetes δ since individuals with diabetes were less likely to be employed compared to those without diabetes, adjusted labor force participation rates for individuals with diabetes and those without were computed in each cohort using the formula: β (α_diabetes_) + (1- β) (α_diabetes_ + δ) = α_population_ [19].

In our baseline scenario, we used estimates from the U.S. Health and Retirement Study which showed that among individuals with diabetes aged 51–61, the absolute probability of working was 4.4 percentage points less for women with diabetes and 7.1 percentage points less for men with diabetes compared to those without diabetes [19]. Hence, δ = 0 was assumed below the age of 51 and is 4.4 and 7.1 % for women and men from 51 to 61 years old respectively in our analysis. Alternative estimates considered but excluded in favor for a gender-specific estimate from our analysis included the U.S. National Health Interview survey which suggested that among persons aged 20–44 years with diabetes, the proportion not working was not statistically significantly different between persons with and without diabetes although the proportion not working among persons aged 45–64 years with diabetes was 8.1 % higher than those without diabetes [20]. On the other hand, the ADA derived an average 2.4 % increase in the likelihood of leaving the workforce for disability from national surveys that increased with age and varied by demographic subgroups from 0.7 percentage points for non-Hispanic white males aged 65–69 years to 7.4 percentage points for non-Hispanic black females aged 55–59 years [12].

### Cost estimation

In this study, the cost analysis was performed based on a conservative approach from a societal perspective (which includes analysis of direct and indirect costs for all stakeholders) from 2010 to 2050 on a per patient basis and projected to the overall Singapore population. Annual and long-term indirect and excess direct costs were expressed in real terms and conversion was done for year 2010 where 1 USD = 1.36 SGD then projected to year 2050 [21]. In this study, we concentrated more on indirect cost as the excess direct cost of a patient with diabetes with diabetes compared to one without has been published previously [22].

#### Estimate of excess direct costs attributable to diabetes

The ADA methodology includes the total medical costs incurred by a diabetes patient as part of the economic burden of diabetes. However, this by definition includes any medical costs that are unrelated to diabetes or its complications (such as annual physical examinations) rather than medical costs that are attributable to the disease, and hence will result in higher estimates. In our analysis, we consider only the excess direct cost incurred by a patient with diabetes relative to one without diabetes.

The estimation of excess direct medical cost for type 2 diabetes patients was based on a local prevalence-based cost-of-illness study in 2010 [22]. Data was drawn from an operational disease registry known as the Chronic Disease Management System (CDMS). This registry consists of patients with diabetes who visited any of the three acute hospitals, one national center, nine primary care clinics and three specialty institutes under the National Healthcare Group (NHG). The NHG is the primary public healthcare group for the entire population of the central and western parts of Singapore, covering 60 % of all public sector primary care attendances in 2010 [23]. Patients were classified as having type 2 diabetes if they satisfied at least one of the three criteria: International Classification of Diseases Ninth Revision, Clinical Modification (ICD-9-CM) with diagnostic code of 250 as primary or secondary diagnosis; received treatment for diabetes for 1 year in any NHG institution; or prescribed any anti-diabetic medication [24]. Type 1 diabetes and gestational diabetes were excluded from the analysis.

Direct diabetes-related costs include costs associated with treatment due to hospitalization, emergency visits, outpatient visits, allied health visits and diabetes-related medications [22]. For our baseline, the mean cost of US$1,496.03 as identified by this local study was used in our model for all ages and gender, as it found that age and gender were not independently associated with cost [22]. We do not differentiate diabetes from its complications because we are interested in the excess cost that a patient with diabetes will incur compared to one without diabetes, which implicitly includes the cost of its complications.

#### Cost estimation of absenteeism

Diabetes increases the likelihood of workdays lost [19]. In this model, the productivity loss was computed using a standard human capital approach, multiplying the estimated workdays lost to diabetes by the total number of workers with diabetes, valued in terms of the average wage for Singaporean residents in 2010 which was US$3,004 [25]. 21 working days per month and 220 workdays a year (after exclusion of personal and public holidays) were assumed in our analysis.

Estimates of workdays lost to diabetes are typically obtained by comparing the absenteeism rates of workers with diabetes to those without. A local study reported an overall mean of 1.3 additional days of medical leave for persons with diabetes compared to those without and the number of workdays lost was not associated with age and gender [26]. The findings were similar to the ADA model which noted an average diabetes-related absenteeism loss of 1.8 days per year per employed person, ranging from 0.9 days for the population aged 18–34 years to 2.5 days for the population aged 45–54 years [2]. In 2012, this average excess absenteeism rate jumped to 3 days for the patients with diabetes [12]. Since local data was available, the value of 1.3 days was used for our baseline analysis.

#### Cost estimation of presenteeism

Presenteeism refers to a decrease in productivity while working [12]. Since a comparable self-report for productivity or a suitable proxy from national surveys was not available in Singapore, the productivity loss figure of 6.6 % (or 14 days per worker with diabetes per year), valued in terms of annual earnings was adopted from the ADA [2].

#### Cost estimation of non-participation in the labor force due to diabetes

Similar to the ADA methodology, the total “missing” workforce due to diabetes, which was defined as the reduction in the workforce due to differences in participation rates for the diabetes population, was estimated [12]. This was done by replicating the hypothetical counterfactual such that the labor force participation rate for diabetes patients was equal to that of those without diabetes in each age cohort in our analysis. For the projection to the year 2050, labor force participation rate was assumed to be constant. The daily cost per person not working was calculated using average gender-specific daily earnings for those working [16]. These earnings were multiplied by a factor of 75 % to account for the generally-observed difference in education levels between those in the workforce and those not in the workforce [2].

#### Cost estimation of non-participation in the labor force due to premature mortality

Mortality results in the permanent loss of an individual’s labor-related income and acquired human capital investments [27]. As with the ADA’s approach, estimates of age and gender-specific mortality rates for the Singapore population were used in this study to calculate the diabetes-attributable deaths from diabetes and diabetes complications like renal disease, cerebrovascular disease, and cardiovascular disease obtained from DEMOS [12]. National productivity loss from early mortality in the case of diabetes-attributable deaths would then be computed by taking the net present value of expected total earnings over the remaining years of life up to the assumed effective retirement age of 69 years old, accounting for the standard probabilities of mortality and nonparticipation as well as changes in wages over the life cycle and general productivity growth. The retirement age in Singapore is 62 years old, up to an age of 65 years old, but we have taken the retirement age to be 69 years old as 30.9 % of the population aged 65–69 years old was still active in the labor force in 2010 [28].

### Future projections using DEMOS model

DEMOS is an individual-level, prevalence-based simulation model that utilizes known risk factors of type 2 diabetes (i.e., age, obesity, ethnicity and genetics), demographic processes (including the mass migration Singapore has experienced over the past two decades), an explicit disease onset submodel, data from national statistics, nationally representative cross-sectional surveys, longitudinal studies, molecular epidemiological cohort studies, as well as the literature to predict the prevalence of diagnosed and undiagnosed type 2 diabetes in Singapore till 2050 via Bayesian statistical methods [13]. Unlike earlier studies, DEMOS does not assume fixed age- and gender-specific prevalence rates [29–31]. In this study, DEMOS was used to predict population size and structure as well as age- and gender-specific diabetes prevalence rates for the Singapore population until 2050, taking migration into account. However, the impact of migration has not been separately quantified in this study as we do not have an alternative null model that excludes migration. DEMOS has been described in detail in previous publications [13].

The economic structure of the population as described below was then applied in each demographic subgroup. In this analysis, productivity growth per annum was assumed conservatively to be 2 % based on Singapore’s national target of 2–3 % annual productivity growth from 2010 to 2020 [32]. Wage growth per annum was assumed to increase at the same rate as productivity growth per annum for the baseline analysis. All analyses were performed in R version 3.0 [33].

### Sensitivity analyses

Parameter uncertainty was explored with various one-way sensitivity analyses. We varied a single parameter between the upper and lower 95 % confidence limits where available; else, ranges were based on literature review or expert opinion if both were not available. Productivity growth of 1 % per annum was from historical trends while 3 % per annum was based on Singapore’s national target in the next decade [32, 34]. The range for wage growth followed that for productivity growth since we assumed both to be in tandem. The range for the number of workdays lost was attained from a local study [26]. The lower end of excess direct medical cost for diabetes was obtained from the mean cost of a diabetes patient without inpatient visit while the upper end of excess direct medical cost was determined from the mean cost of a diabetes patient with more than one inpatient visits [22]. The number of diabetes patients, individuals without diabetes and diabetes-related deaths were based on 95 % CIs obtained from DEMOS while the remaining parameters (diagnosed prevalence of diabetes, productivity gap as well as number of workdays lost due to presenteeism) were assigned a range of low to high values based on expert opinion [13].

## Results

### Baseline cost estimation

**Fig. 1:**
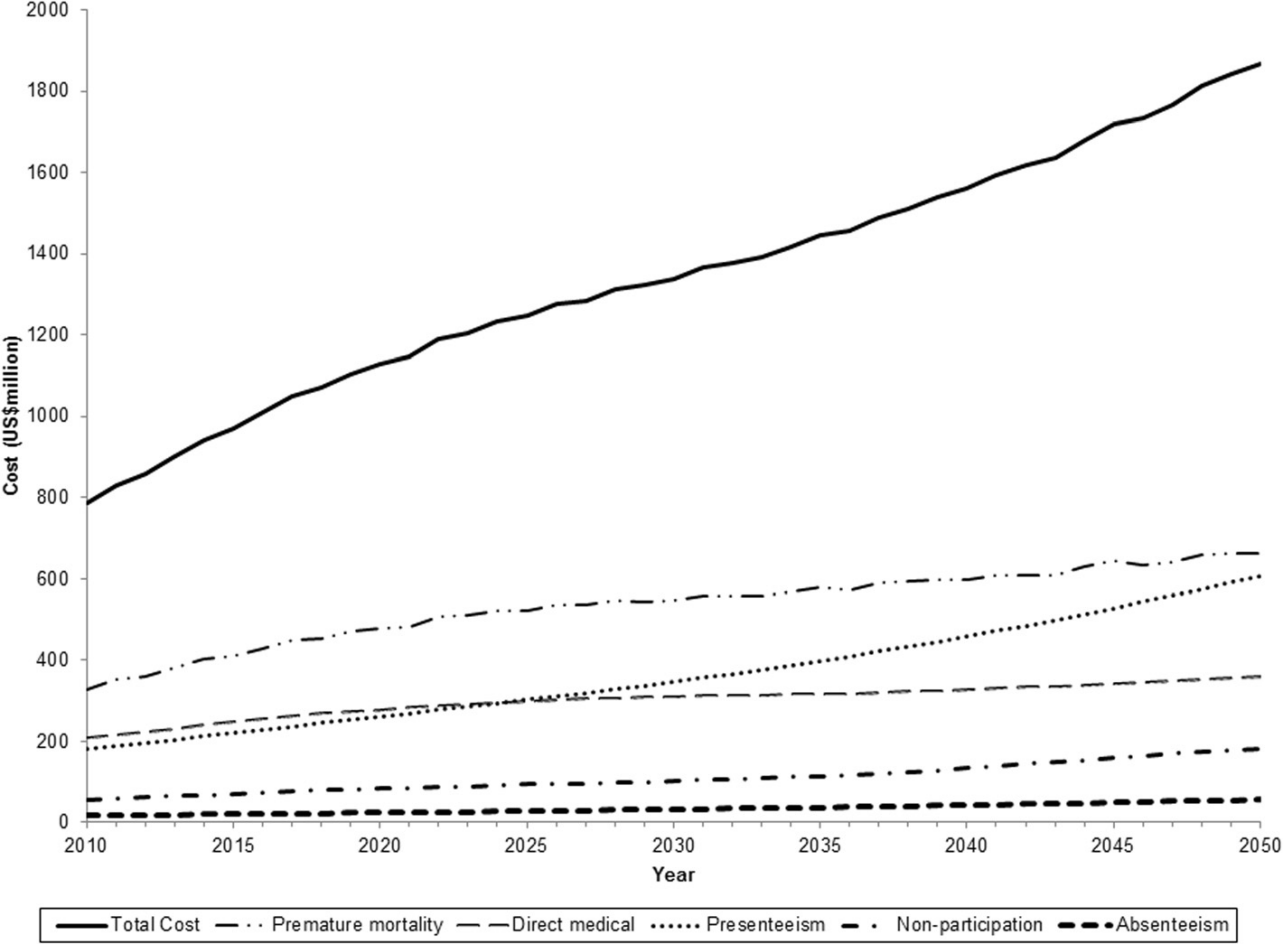
Total economic burden of working-age patients with diagnosed type 2 diabetes in Singapore from 2010 to 2050

In 2010, the estimated total economic cost for working-age type 2 diabetes patients in Singapore in our baseline scenario is US$787 million (58 % indirect cost and 42 % direct medical cost; cost of absenteeism: US$17 million, cost of presenteeism: US$180 million, cost of lost capacity from premature mortality: US$327 million and cost of diabetes-related non-participation: US$54 million). This figure is projected to increase to US$1,867 million in 2050 (65 % indirect cost and 35 % direct medical cost; cost of absenteeism: US$56 million, cost of presenteeism: US$607 million, cost of lost capacity from premature mortality: US$663 million and cost of diabetes-related non-participation: US$182 million) as seen in Fig. 1. The total economic cost per patient in this population is therefore estimated to be US$5,646 in 2010 and US$7,791 in 2050.

Tables 1 and 2 depict the costs per diagnosed type 2 diabetes patient in the local working-age population by age groups from 2010 to 2050 for males and females respectively. Male working-age type 2 diabetes patients incurred a higher total economic cost than their female counterparts. In addition, the total economic burden of a patient with type 2 diabetes was the highest among those aged 50–59 years old at US$8,574 for males and US$4,602 for females in 2010 and this would increase to US$13,170 and US$6,524 for males and females in 2050 respectively.

**Table 1:** Breakdown of different costs per diagnosed type 2 diabetes patient in the Singapore male working-age population by age group from 2010 to 2050 (in USD)

Year	Age group	Diabetes population	Indirect productivity loss					Total economic cost^a^
			Absenteeism	Presenteeism	Diabetes-related non-participation	Lost capacity from premature mortality	Total indirect cost	
2010	20–29	2,396	150	1,614	-	-	1,764	3,260
	30–39	8,205	260	2,795	-	1,874	4,929	6,425
	40–49	19,334	229	2,464	-	3,428	6,121	7,617
	50–59	27,760	144	1,555	1,270	4,109	7,078	8,574
	60–69	21,114	67	722	240	2,340	3,369	4,865
2030	20–29	2,920	225	2,426	-	394	3,046	4,542
	30–39	10,149	386	4,154	-	1,974	6,513	8,009
	40–49	22,004	340	3,663	-	3,821	7,824	9,320
	50–59	35,009	213	2,296	1,882	5,256	9,648	11,144
	60–69	50,298	95	1,022	229	2,461	3,806	5,302
2050	20–29	3,238	340	3,657	-	-	3,996	5,492
	30–39	12,013	573	6,172	-	2,520	9,265	10,761
	40–49	25,323	505	5,442	-	4,512	10,459	11,955
	50–59	42,216	318	3,422	2,748	5,186	11,674	13,170
	60–69	58,576	142	1,534	394	2,326	4,397	5,893

^a^Total economic cost = Total indirect cost + Excess direct medical cost of US$1,496.03

**Table 2:** Breakdown of different costs per diagnosed type 2 diabetes patient in the Singapore female working-age population by age group from 2010 to 2050 (in USD)

Year	Age group	Diabetes population	Indirect productivity loss					Total economic cost^a^
			Absenteeism	Presenteeism	Diabetes-related non-participation	Lost capacity from premature mortality	Total indirect cost	
2010	20–29	1,451	122	1,313	-	-	1,435	2,931
	30–39	5,286	157	1,693	-	430	2,280	3,776
	40–49	13,520	119	1,285	-	1,572	2,976	4,472
	50–59	21,367	66	714	568	1,758	3,106	4,602
	60–69	18,956	19	210	85	1,115	1,429	2,925
2030	20–29	2,028	182	1,955	-	-	2,137	3,633
	30–39	6,954	234	2,521	-	577	3,332	4,828
	40−49	14,073	177	1,911	-	1,855	3,943	5,439
	50–59	24,739	97	1,046	853	2,123	4,118	5,614
	60–69	39,338	27	291	83	1,288	1,689	3,185
2050	20–29	2,180	273	2,937	-	-	3,210	4,706
	30–39	7,957	348	3,746	-	641	4,735	6,231
	40–49	17,070	264	2,840	-	2,007	5,110	6,606
	50–59	29,813	145	1,562	1,247	2,073	5,028	6,524
	60–69	41,261	41	436	144	1,494	2,115	3,611

^a^Total economic cost = Total indirect cost + Excess direct medical cost of US$1,496.03

### Sensitivity analyses

**Fig. 2:**
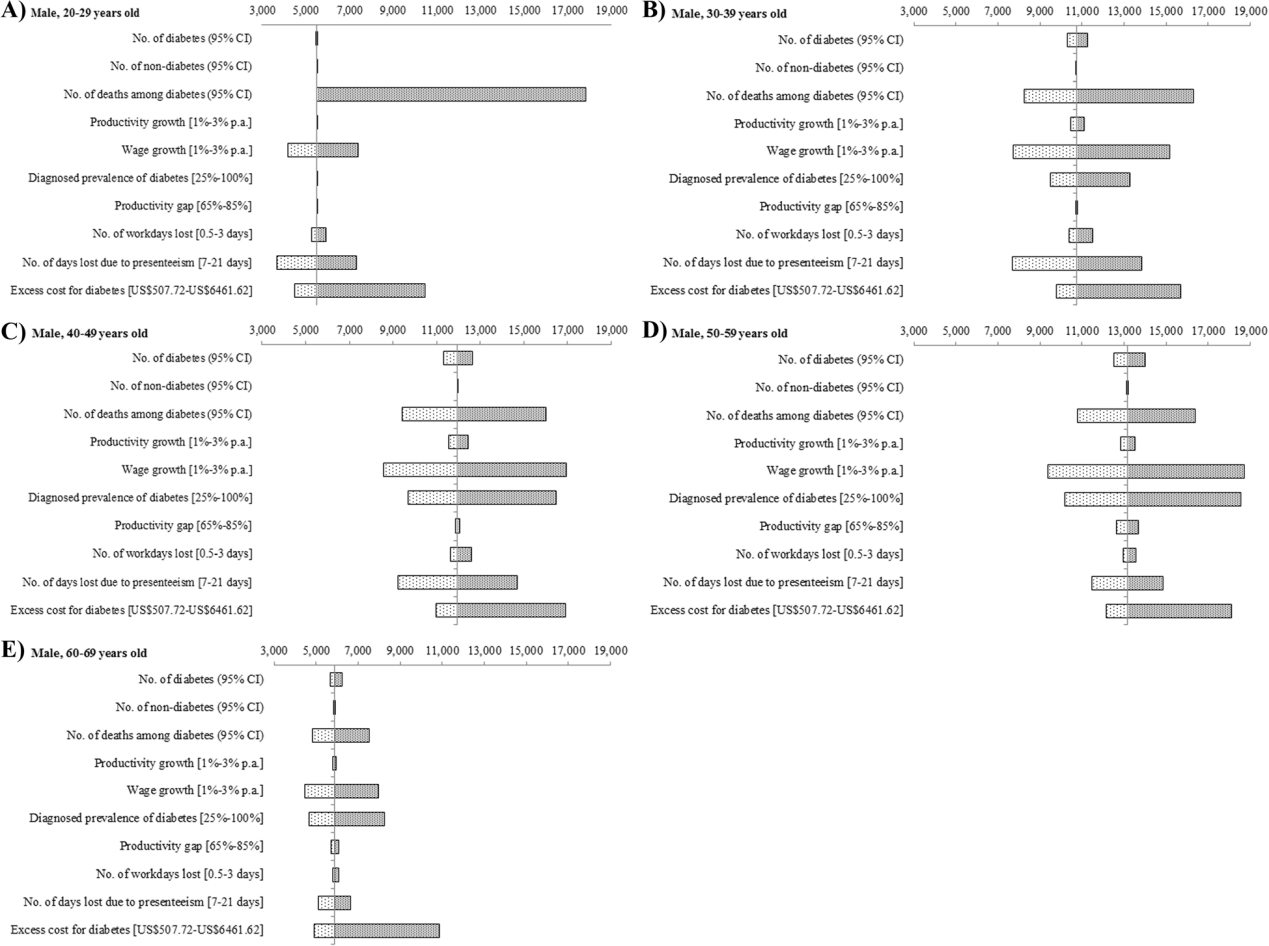
Tornado diagrams of one-way sensitivity analyses among males with diagnosed type 2 diabetes by age groups in 2050. Range of total cost per diagnosed type 2 diabetes patient associated with range of each parameter indicated in brackets (in USD)

Sensitivity analysis applied to total cost per patient in 2010 would result in cost between US$4,432 to US$10,612 whereas in 2050, the total cost per patient would be between US$5,741 and US$12,756. Figure 2 shows the sensitivity analyses for working-age males by age groups in 2050. Results from working-age females showed the same trend albeit at a lower increment and so were not presented separately in this article. Among those 20–39 years old, the number of deaths among diabetes was the most influential on the total cost per patient in 2050. The total cost per patient among 20–29 years old would increase up to US$12,361 from base case estimate while those among 30–39 would increase up to US$5,533 and decrease up to US$2,520 from base case estimate. Wage growth was the most influential parameter on total cost per patient in 2050 for those aged 40–59 years old. The total cost per patient among 40–49 years old would grow up to US$4,993 and reduce up to US$3,407 from base case estimate while those among 50–59 would grow up to US$5,572 and reduce up to US$3,802 from base case estimate. Lastly, for those aged 60–69, the excess direct medical cost of diabetes was the most influential on total cost per patient in 2050 with cost that would increase up to US$4,966 and reduce up to US$988 from base case estimate.

## Discussion

Despite the growing epidemic of type 2 diabetes, to our knowledge, this study is the first to predict the diabetes-related social cost of the working-age population not just for Singapore, but possibly for any country in Asia from 2010 to 2050 [35]. In 2010, Singapore’s GDP was US$223 billion, bringing the total diabetes-related economic cost among the working-age population to about 0.35 % of Singapore’s GDP [36]. Considering that Singapore currently spends only 4 % of its GDP on healthcare expenditure, even in our conservative scenario, diabetes has imposed a significant economic burden on the national health care system and will continue to do so in the next four decades [37].

Among this population, indirect costs are a significant driver of the total burden, and will increase in importance over time. Working-age males, especially those aged 50–59 in our analysis, were found to incur a higher total economic cost than females primarily because of the higher labor force participation rate of males in the workforce and a higher income than their female counterparts in the same age group [16].

The results also showed that projected trends in healthcare spending are likely to continue, both in terms of total cost as well as the shifting emphasis on indirect costs. This suggests that prevention efforts are relevant not only to policymakers in the health sector, but also to policymakers and employers concerned with labor force productivity who will bear the “unseen” majority of diabetes cost in the future.

Finally, this study illustrates that differences in underlying disease and cost drivers as well as methodology can complicate comparisons across time. Although we have largely adopted the ADA methodology in our analysis, direct comparisons are not easily facilitated as our findings vary for both methodological and substantive reasons [12]. For instance, firstly, Singapore’s total cost estimate of 0.35 % of GDP is low relative to the ADA estimate of 1.52 % for the U.S. [38], and in the U.S., 72 % of the total economic burden for diabetes patients is direct medical costs, compared to 42 %. However, this disparity is partly due to the use of total versus excess direct medical costs, and also due to different levels of healthcare cost in Singapore versus the U.S. Secondly, excluding costs due to disability which were not available for Singapore, presenteeism was the highest cost driver in the U.S. [12]. This trend was not observed in our study where the cost of lost capacity from premature mortality was the highest contributor, possibly due to the due to a higher proportion of older diabetes patients with an increasing mean age (58.2 years old in 2010 to 66.9 years old in 2050) and a higher average wage compared to other age groups.

There are several important limitations to this study, some of which are inherent to the ADA methodology which was adapted for comparability. For example, the methodology used to estimate the lost productivity from early mortality due to diabetes is not in concept consistent with the costing for the other components (which represent actual flows realized during the year of accounting) and gives estimates that are extremely sensitive to the assumptions. Ideally, productivity loss from premature mortality should be computed as the foregone value of productivity in a given year from individuals who died from diabetes in all prior years but would otherwise have been alive. However, the ADA (and our research team) did not implement this approach due to practical difficulty. This could yield very significant differences in calculated losses from diabetes attributed mortality from small changes in the discount rate applied. Most critically, the analysis relies on assumptions which were drawn from multiple sources, a number of which are based on assumptions adopted in the U.S. (and used in other international settings). Unfortunately, locally available primary data or secondary research are not yet sufficiently well developed in most cases to supply the necessary parameters for our analysis. We have addressed these limitations by estimating alternative scenarios where the data are available. In our baseline scenario, we adopt a consistently conservative approach, such that our figures may be viewed as lower bounds for costs. For example, the undiagnosed rate for diabetes was assumed to be constant in our analysis due to conflicting trends projected in literature [1, 6, 39]. In addition, the assumption for excess direct medical cost here was it did not differ by age and gender based on results from a local study [22] despite two previous studies which suggested otherwise [40, 41]. Furthermore, we did not include the economic burden of those with undiagnosed diabetes as well as the cost of informal care which may contribute to a significant portion of indirect cost as observed by Hex et al. [5]. This would in particular underestimate the cost of diabetes for women as previous studies have found that informal care is typically provided by women [42].

In addition to being the first analysis of this kind for Asia to our knowledge, a key strength of the study is the generation of age- and gender-specific estimates, allowing us to assess the evolution of sub-group differences. A further key strength is the ability to assess the relative importance of the different indirect cost components. Both of these features allow for more nuanced policy interpretations for all stakeholders (including those outside the health sector) such as prioritizing the need to target specific groups, or specific interventions (e.g., reducing workplace presenteeism or addressing medical costs). Finally, in principle, this methodology can be replicated in other countries as well to generate regional estimates.

Work in progress in Singapore includes the gathering of disaggregated cost data (e.g., by ethnic and socioeconomic groups) to enable more accurate estimates of diabetes-related expenditures due to complications; obtaining better estimates of diabetes-related mortality and incorporating the comparative cost effectiveness of interventions like drug interventions or active lifestyle programmes into the model [43]. Planned future work includes the collection of population-based data to allow better estimation of the effects of diabetes on employment and productivity. The inclusion of this data as well as the development of these improved estimates would help enhance the accuracy of the cost estimations presented in this study as well as help policymakers anticipate future costs of diabetes and determine effective public health interventions.

## Conclusions

This study shows that the considerable rising economic burden of diabetes in this setting will affect not just individuals and health providers but also employers and society overall through the impact of lost productivity. Coupled with earlier onset and population ageing, the burden on Asian countries in the future is likely to be substantial if these forecasts were proven to be accurate.
