# Erratum to: ‘Current and future economic burden of diabetes among working-age adults in Asia: conservative estimates for Singapore from 2010-2050’

**DOI:** 10.1186/s12889-016-3164-0

**Published:** 2016-07-18

**Authors:** May Ee Png, Joanne Yoong, Thao Phuong Phan, Hwee Lin Wee

**Affiliations:** National University of Singapore, Saw Swee Hock School of Public Health, Singapore, Singapore; University of Southern California, Center for Economic and Social Research, Los Angeles, USA; National University of Singapore, Department of Pharmacy, Singapore, Singapore

Unfortunately, the original version of this article [1] contained an error. After publication the authors became aware of the mistakes in computing the proportions of excess direct medical costs and indirect productivity-related losses in the *Abstract, Results* and *Discussion* (described as follows). The proportion of excess direct medical costs and indirect productivity-related losses in 2010 were updated from 42 to 26 % and 58 % to 74 % respectively. Likewise, the share of indirect costs in 2050 rose to 81 % instead of 65 % as previously written with the direct medical cost reduced to 19 % instead of 35 %. In the *Discussion* section, in the U.S., 72 % of the total economic burden for diabetes patients is direct medical costs, compared to 26 % instead of 42 % in Singapore.

In making these changes we became aware of two other errors that had not been corrected at final draft stage of the publishing process. These are described below:

i. Under the sub-section *Cost estimation of absenteeism* from *Methods* section, the average wage for Singaporean residents in 2010 that was used in the analysis was US$3,446 for males and US$2525 for females instead of US$3,004.
ii. The values in the *Sensitivity analyses* sub-section from *Results* section were the incremental differences from baseline values. Hence, the paragraph should read: The total cost per patient among 20–29 years old would increase by up to US$12,361 from base case estimate while those among 30–39 would increase by up to US$5,533 and decrease by up to US$2,520 from base case estimate. Wage growth was the most influential parameter on total cost per patient in 2050 for those aged 40–59 years old. The total cost per patient among 40–49 years old would grow by up to US$4,993 and reduce by up to US$3,407 from base case estimate while those among 50–59 would grow by up to US$5,572 and reduce by up to US$3,802 from base case estimate. Lastly, for those aged 60–69, the excess direct medical cost of diabetes was the most influential on total cost per patient in 2050 with cost that would increase by up to US$4,966 and reduce by up to US$988 from base case estimate.

None of the conclusions reached in the uncorrected version of the paper has been revised in the light of these changes.
